# Intraoral Scanners in Orthodontics: Utilization, Awareness, and Educational Implications Among Specialists in the Kurdistan Region, Iraq – A Cross-Sectional Study

**DOI:** 10.1155/ijod/6663009

**Published:** 2025-06-04

**Authors:** Salar Karim Khalil, Mohamad Radwan Sirri

**Affiliations:** ^1^Department of Medical Laboratory Technology, Technical Institute of Zakho, Duhok Polytechnic University, Duhok, Iraq; ^2^Department of Orthodontics, University of Damascus Dental School, Damascus, Syria

**Keywords:** awareness, clinical training, digital dentistry, educational challenges, intraoral scanners, orthodontics

## Abstract

**Objectives:** Intraoral scanners (IOS) are revolutionizing orthodontics by providing advanced digital tools for diagnosis and treatment. However, limited information exists on their utilization, awareness, and educational impact among orthodontists in the Kurdistan region of Iraq. This study evaluates IOS adoption, technical proficiency, and challenges to address educational gaps and enhance clinical integration.

**Materials & Methods:** A cross-sectional online survey was distributed to 385 orthodontists in Erbil, Duhok, Sulaymaniyah, and Zakho. The 31-item questionnaire assessed demographics, experience, awareness, technical knowledge, clinical applications, and perceived challenges. Data collected from April to August 2024 were analyzed using SPSS v26.0, incorporating descriptive statistics, correlation analysis (Pearson's/Spearman's), and gap analysis.

**Results:** Among 290 respondents (75.32% response rate), 61.03% used IOS, though 58.89% lacked technical expertise. Most participants lacked formal academic training, with a majority self-rating as moderately skilled. Younger practitioners adopted IOS more frequently (Pearson's *r*=−0.42, *⁣*^*∗*^*p*^*∗*^=0.002), while older specialists faced greater challenges (Spearman's *ρ*=0.38, *⁣*^*∗*^*p*^*∗*^=0.021). Private-sector adoption surpassed public-sector use (*ρ*=0.65, *⁣*^*∗*^*p*^*∗*^ < 0.001). Primary applications included aligners (46.20%) and digital archiving (22.76%), with underutilized diagnostic potential (5.52%). Key barriers were training shortages (60.34%) and high costs (35.17%).

**Conclusions:** Critical gaps in IOS adoption persist, including disparities in technical knowledge, generational proficiency, sectoral access, and clinical application. Addressing these requires integrating IOS training into academic programs, hands-on workshops, subsidized access for public practitioners, and industry-backed certifications. Collaboration between universities and manufacturers is key to standardizing expertise and improving patient outcomes.

**Summary**

• Orthodontists in the Kurdistan region of Iraq are increasingly adopting intraoral scanners (IOS); however, they often lack formal education on the technical aspects.
• While most specialists acknowledge the benefits, there is a significant need for tailored training programs and curriculum development to enhance proficiency.
• This study highlights the importance of addressing the educational gap to maximize the utilization of this technology in orthodontics.

## 1. Introduction

The field of dentistry has undergone a profound digital transformation, driven notably by intraoral scanners (IOS) [1]. This shift began with CAD/CAM systems in 1973 [2], accelerated by Sirona's CEREC in 1987 [3], and reached a turning point in 2008 with Cadent iTero's first commercial IOS [4]. IOS devices now offer trueness, precision, resolution, scanning speed, tip design, color imaging, and system integration, revolutionizing workflows across specialties [5].

IOS technology serves critical roles across dental specialties [5]. Prosthodontics enables the creation of crowns, bridges, and dental prostheses, including digital smile design [6]. In implantology, it ensures precision in guided implant surgery planning [7]. For orthodontics, IOS helps diagnose arch dimensions and tooth discrepancies [8]. It also supports orthognathic surgery planning and streamlines workflows like aligner production, custom-made devices, and indirect bonding [9].

Although IOSs enhance patient comfort, time efficiency, streamlined procedures, better communication, rapid data access, and space savings [10], challenges persist. These include difficulty detecting deep marginal lines, a steep learning curve, and high acquisition and management costs. Consequently, only about 20%–25% of European dental offices have adopted IOS technology [11].

Research on IOS in dentistry is widespread, but most studies do not fully assess students and general dentists' understanding of IOS features, benefits, or practical use [12]. This gap becomes clearer when comparing general dentists to specialists in how they adopt or view IOS technology [11].

Existing literature highlights varied global and regional trends in IOS adoption. For instance, Al-Hassiny [11] et al.'s multinational survey of dentists in 109 countries found that 78.8% of dentists use IOS daily, primarily Medit and Dentsply Sirona, with cost and speed as key barriers despite 81.9% endorsing IOS accuracy over traditional methods. Regionally, In Saudi Arabia, Al-Qahtani et al. surveyed 500 practitioners and students, revealing 58% awareness but 43.5% nonuse of IOS, with cost as the primary obstacle (57.39% males, 50.86% females) and workshops as the preferred training method [13]. Hall et al. [14] surveyed 402 Egyptian dentists, noting moderate knowledge (47.3%) but high perceived value (75.9%), with urban specialists outperforming rural peers, though orthodontist perspectives were absent. In the Netherlands, Van der Zande et al. [15] surveyed 249 general dental practitioners, revealing that administrative technologies (e.g., digital patient records) were more widely adopted (93.2%) than clinical tools, like IOS (12%), with high-tech users being younger, specialized, and working in larger practices. In contrast, Nakornnoi et al. [16] assessed 112 Thai orthodontic residents, who ranked IOS highest in importance (4.37/5) and confidence (4.23/5), with 87.5% advocating for mandatory training integration.

Despite these insights, no study has specifically examined orthodontic specialists' proficiency, awareness of IOS's diagnostic/therapeutic potential, or adoption challenges—a critical oversight in developing regions, like the Kurdistan region of Iraq, where technological integration remains underexplored.

To address this gap, the current study targets orthodontic specialists in Iraq's Kurdistan region, including public and private sectors across major centers (Erbil, Duhok, Sulaymaniyah, and Zakho). The research evaluates specialists' familiarity with IOS, proficiency in using the technology, perceived clinical utility, and key challenges. By identifying barriers and opportunities, the findings aim to provide actionable insights for optimizing IOS integration into orthodontic practice, enhancing its effectiveness and accessibility for specialists.

### 1.1. Research Objective and Hypothesis

This study addresses the following central research question: What are the utilization rates, awareness levels, and educational challenges associated with IOS adoption among orthodontic specialists in the Kurdistan region of Iraq? Based on the identified gaps in existing literature, the hypotheses are:

• A significant gap exists between clinical adoption of IOS and technical proficiency, influenced by generational disparities and sectoral divides (public versus, private).
• Limited academic training and financial barriers hinder full integration of IOS into routine practice, despite its perceived clinical advantages.

By investigating these dimensions, the study aims to bridge the knowledge gap and provide evidence-based recommendations to optimize IOS adoption, enhance clinical training, and improve accessibility in resource-limited settings.

## 2. Materials and Methods

### 2.1. Study Design and Setting

This descriptive cross-sectional study targeted orthodontic specialists practicing in the Kurdistan region of Iraq (Erbil, Dohuk, Sulaymaniyah, Zakho). Eligible participants held a master's degree or advanced clinical certification in orthodontics. Submissions with incomplete responses or practitioners lacking advanced qualifications were excluded. Ethical approval was obtained from the Research Ethics Committee at Duhok Polytechnic University (Approval no. KR-8851-2024/DPU5521), adhering to the Declaration of Helsinki. The study included orthodontists from the public (universities: *n* = 3, health centers: *n* = 30) and private sectors (private universities: *n* = 5, dental centers: *n* = 125, and clinics: *n* = 218).

### 2.2. Sample Size Calculation

**Figure 1:**
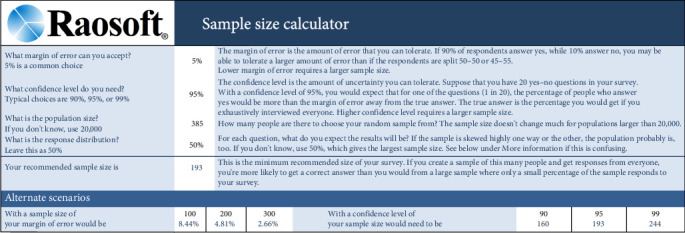
Sample size calculation process using an online tool.

Using Raosoft's online tool (www.raosoft.com), the sample size was calculated as 193 participants (95% confidence level, 5% margin of error), based on a total population of 385 orthodontists registered with the Dental Syndicate and the Ministry of Health in the Kurdistan region of Iraq, as detailed in Figure 1.

### 2.3. Questionnaire Development and Domains

The questionnaire was developed in three phases: (1) Theoretical foundation phase, based on a literature review; (2) validation process phase, including expert review and validity testing (content validity ratio [CVR]/content validity index [CVI]); and (3) reliability assurance phase, which involved pilot testing and Cronbach's alpha coefficient (*α* = 0.85). Full methodological details are presented in the Table S1.

The final questionnaire comprised six domains with 31 questions assessing IOS adoption in orthodontics: (1) Demographics, covering practice setting, and qualifications; (2) IOS experience, including usage frequency and clinical context; (3) awareness and education, focusing on academic training and hands-on exposure; (4) scanner characteristics, addressing technical features and operational protocols (Questions 21–27); (5) system applications, emphasizing clinical and diagnostic uses; and (6) perceived advantages and challenges, such as accuracy and financial barriers. Various question formats, including nominal rankings, multiple-choice, and free-text responses, ensured a balanced analysis. The online survey is accessible here: https://docs.google.com/forms/d/1Rz7XNYayk8n3gwx9s2p8p3prVhKHqGzuKrHU1IkEPJ4/edit.

### 2.4. Questionnaire Administration and Bias Control

The questionnaire was distributed electronically (Google Forms) via email or WhatsApp. An independent researcher managed data collection, entry, and validation to minimize bias. To ensure anonymity and honest responses, all submissions were confidential. Cross-validation questions were included to detect self-reporting bias. All questions were mandatory, clearly specifying single or multiple selections, with only one submission allowed per participant. Clarifications were provided as needed, and nonrespondents received a reminder after 2 weeks. Data collection took place between April and August 2024.

### 2.5. Statistical Analysis

Data were coded and analyzed using Statistical Package for the Social Sciences (SPSS) version 26.0 (IBM, USA). The Shapiro–Wilk test confirmed a normal distribution for quantitative variables (years of experience, IOS usage frequency, and number of IOS devices) (*p*  > 0.05), allowing the use of parametric tests. Descriptive statistics, including percentages and frequency distributions, were visualized with bar and pie charts. Pearson's correlation analyzed linear relationships between continuous variables, while Spearman's correlation was used for ranked data. Multiple linear regression identified factors influencing IOS adoption and a gap analysis compared academic knowledge with clinical practice.

## 3. Results

**Figure 2:**
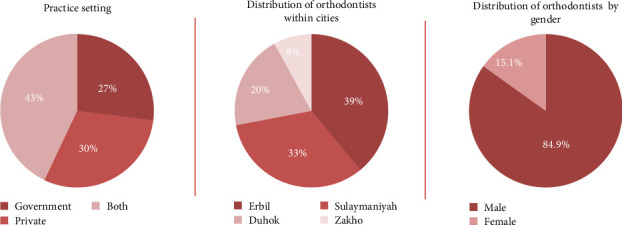
Distribution of orthodontists by practice setting, cities, and gender.

**Figure 3:**
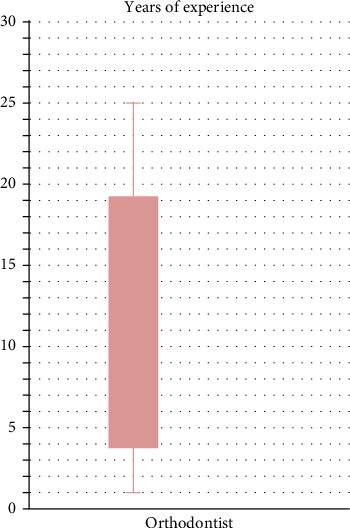
Years of experience.

**Figure 4:**
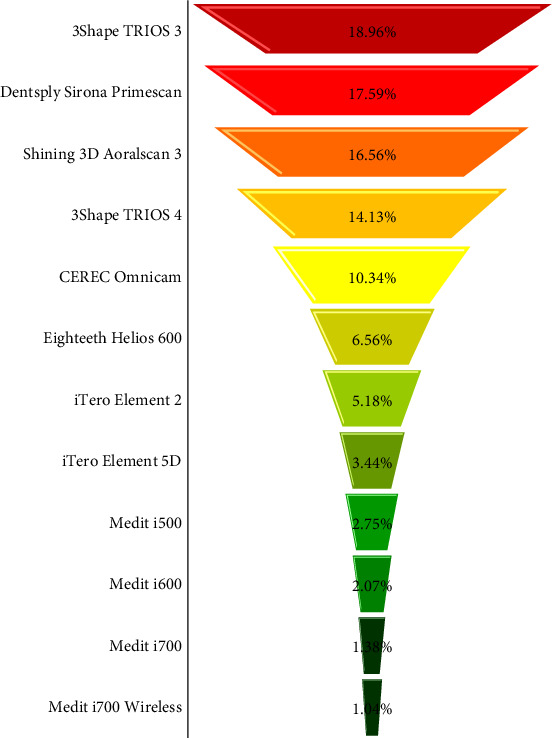
The most common brand of IOS used.

A survey was sent to 385 orthodontic specialists in the Kurdistan region, yielding a 75.32% response rate (*n* = 290). Most respondents were male (84.9%), and 43% worked in both public and private sectors, with clinical experience ranging from 1 to 25 years (Figures 2 and 3). IOS was used by 61.03% of orthodontists, 81.03% owned at least one scanner, and 38.96% used it monthly. While 75.17% had moderate experience, only 2.76% were experts. Most (79.31%) found IOS more accurate than traditional methods, and 91.37% expected further precision improvements. Free software accounted for 92.41% of use, with paid versions representing just 2.06% (Table 1). Among scanners, the 3Shape Trios 3 led the pack at 18.96% (Figure 4).

**Figure 5:**
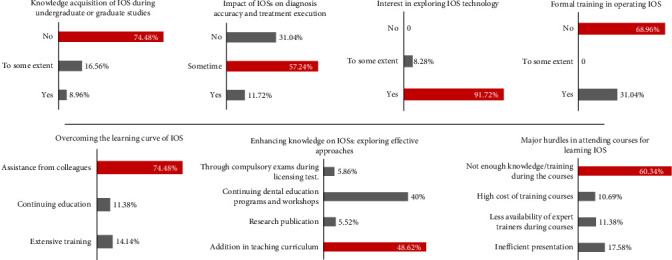
Enhancing awareness and education.

Most participants (74.48%) lacked IOS academic training during their studies, yet 57.24% recognized its diagnostic and treatment execution potential. Despite a strong interest in the technology (91.72%), 68.96% lacked formal training in its operation. Peer assistance (74.71%) was a key learning source, with preferred training methods including curriculum additions (48.62%) and dental programs (40%). Challenges included insufficient training (60.34%) and ineffective presentations (17.58%) (Figure 5).

**Figure 6:**
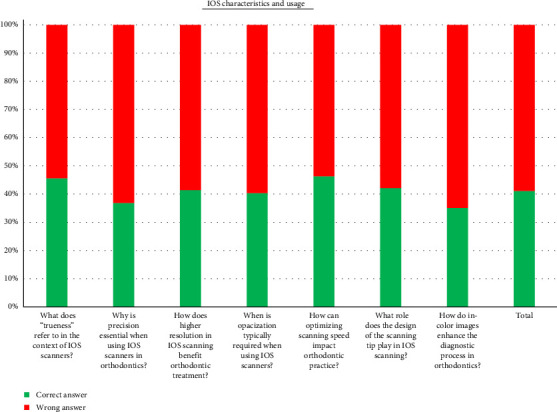
Scanner characteristics and usage.

**Figure 7:**
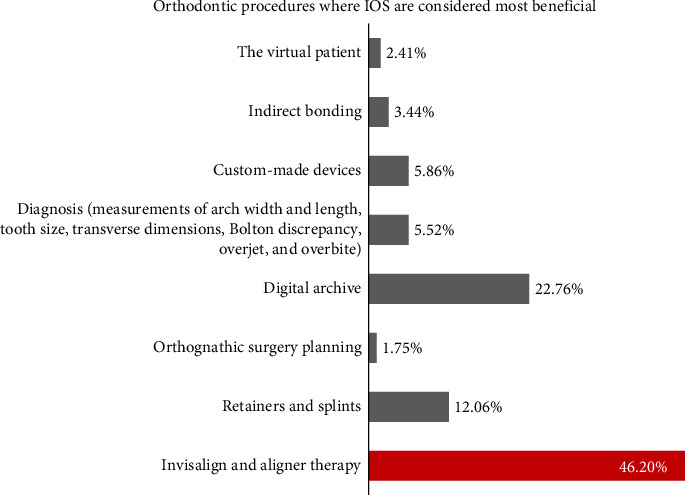
System applications.

Regarding scanner usage, 58.62% of responses to technical questions were incorrect (Figure 6). IOS was primarily used for clear aligners (46.20%) and digital archiving (22.76%) (Figure 7). Advantages included improved patient experience (48.96%), high accuracy (28.62%), and scanning speed (24.82%), while the main drawback was high initial cost (35.17%) (Table 2).

Correlation analysis revealed that younger age was associated with higher IOS usage (Pearson's *r*=−0.42, *p*=0.002), while public sector work was associated with greater knowledge gaps (Spearman's *ρ*=0.65, *p*  < 0.001). Academic training enhanced clinical reliance on IOS (Spearman's *ρ*=0.55, *p*  < 0.001), and owning more devices reduced work challenges (Pearson's *r*=−0.45, *p*=0.003). Older practitioners faced more technological challenges (Spearman's *ρ*=0.38, *p*=0.021) (Table 3).

A multiple linear regression analysis identified key factors affecting IOS adoption and usage (Table 4). Formal IOS training had the strongest positive impact on clinical use (*β*=0.67, *p*=0.01). Owning more IOS devices was linked to higher monthly usage (*β*=0.45, *p*=0.003), while greater expertise increased confidence in digital accuracy (*β*=0.55, *p*  < 0.001). Free software upgrades (*β*=0.32, *p*=0.04) and private sector employment (*β*=0.32, *p*=0.03) also promoted adoption.

The gap analysis revealed several key gaps related to using IOS (Table 5). The experience gap was evident, as IOS was widely used clinically despite insufficient formal training, leading to superficial adoption and limited proficiency. The educational gap reflected inadequate academic preparation, which reduced technical competence and confidence in diagnostic use. The technical understanding gap indicated an overreliance on digital scanning accuracy without a solid grasp of technical terms, potentially affecting clinical decision-making. The clinical application gap showed a preference for cosmetic use over diagnostic potential, limiting the full benefits of IOS in practice. The structural challenges gap involved insufficient training and high costs, which hindered the widespread and effective adoption of the technology.

## 4. Discussion

Despite the widespread adoption of IOS technology in dentistry [17], data on its use among orthodontic specialists remains limited [8, 18]. This study is the first to comprehensively survey orthodontists in Erbil, Sulaymaniyah, Dohuk, and Zakho, offering insights into their advantages and challenges. The findings enhance understanding for both practitioners and manufacturers, guiding future research and investment decisions.

### 4.1. IOS Adoption Gaps in Orthodontic Practice: Age and Sector Related Disparities

The study found that 61.03% of orthodontists use IOS, similar to Al-Qahtani et al. [13] (56.5% of participants) but lower than Al-Hassiny et al. [11] (78.8% of participants), reflecting a shift toward digital dentistry. Higher IOS ownership led to frequent use. While 38.96% of orthodontists used IOS monthly, 49.5% of participants in Al-Hassiny et al.'s study used it daily [11], likely due to a more diverse sample.

A generational gap was observed in IOS adoption. Younger practitioners used IOS more frequently, while older practitioners faced challenges. Studies by Alqahtani et al. [13], Nakornnoi et al. [16], and Van der Zande et al. [15] confirmed that younger dentists are more interested in IOS but lack proper training, underscoring the need for better integration into dental curricula.

Private clinics had higher adoption rates due to greater financial flexibility, while public healthcare faced budget constraints, as noted by Nakornnoi et al. [16]. Van der Zande et al. [15] and Al-Hassiny et al. [11] also noted that better infrastructure in private clinics accelerates IOS adoption.

To address these inequities, strategic public-private partnerships and tailored educational programs for public-sector and rural practitioners, as proposed by Hall et al. [14], are critical to enhancing digital literacy, optimizing resource distribution, and reducing knowledge gaps.

### 4.2. The Gap Between Theoretical Knowledge and Practical Application

The findings emphasize a critical disconnect between theoretical awareness and clinical application of IOS, exacerbated by insufficient formal education. Although most orthodontists had adopted IOS in their daily practice, their technical proficiency remained shallow because they had relied primarily on informal training. Furthermore, although diagnostic potential was acknowledged, its consistent clinical utilization was limited. Limited curriculum integration forces many to rely on informal training. Similarly, Alqahtani et al. [13] found that 58% were aware of IOS, yet 43.5% did not use it. In contrast, Schott et al. [19] showed that intensive training improves proficiency, and Nakornnoi et al. [16] indicated that 87.5% of orthodontic residents support mandatory IOS training.

The analysis underscores the pivotal role of academic training in enhancing clinical performance with IOS. Addressing this gap necessitates curriculum reforms, including mandatory hands-on modules and competency assessments to ensure sustained skill development

### 4.3. The Technical Knowledge Gap

The study identified a gap in technical knowledge. This gap points to problems in formal education and overreliance on informal learning, suggesting the urgent need for standardized training frameworks. Public-sector orthodontists showed larger gaps than their private-sector peers, likely due to fewer resources and support.

To address these challenges, competency-based training should be added to education, as recommended by Nakornnoi et al. [16] and Schott et al. [19], along with specialized programs for public-sector staff, supported by Alqahtani et al. [13] and Van der Zande et al. [15], to improve skills and access. Public-private partnerships, as noted by Al-Hassiny et al. [11], could also boost knowledge sharing and equal access to IOS technology

### 4.4. The Gap Between Actual and Intended Use: Challenges and Solutions

The results revealed a discrepancy between the “**actual use**” of IOS technology and its “**intended use**” (as designed by manufacturers). Specifically, the technology is primarily utilized for designing clear aligners and digital archiving, while neglecting its diagnostic aspects. These findings corroborate the studies by Khalil et al. [18] and Frąckiewicz et al. [20], which demonstrated that many practitioners have not yet leveraged the advanced diagnostic capabilities of this technology.

Despite its accuracy in treatment planning, IOS's diagnostic potential remains underutilized. Khalil et al. [18] and Frąckiewicz et al. [20] revealed that many orthodontists have yet to fully integrate these features into clinical practice. Al-Hassiny et al. [11] and Spangnuolo and Sorrentino [21]. identified this gap, emphasizing the need for targeted education. Spangnoulo et al. also stressed the importance of aligning IOS features with clinicians' practical needs for better integration.

IOS enhances patient experience by reducing discomfort, consistent with Christophelou et al. [10]. High accuracy is supported by advancements in AI and image-stitching, consistent with Eggmann and Blatz [22], though challenges persist in severe crowding and edentulous areas. Jedliński et al. [9] Note 30% faster workflows, though skill-dependent. High initial costs hinder adoption, yet long-term savings via open-source systems and reduced materials are possible [22]. Further research is needed for affordable models, complex-case accuracy, and optimized training to maximize IOS potential.

## 5. Limitations

The questionnaire-based approach, while efficient, may not fully capture participants' experiences, suggesting the need for qualitative research. Its cross-sectional design also prevents tracking long-term trends, making longitudinal studies essential. Although selection bias was minimized through diverse recruitment, voluntary response bias may persist, as orthodontists familiar with IOS were more likely to participate. Additionally, reliance on self-reported data may introduce reporting bias, such as overestimation of IOS usage or underestimation of challenges due to social desirability. For instance, practitioners in competitive private sectors might overstate their technical expertise, while public-sector professionals might underreport financial constraints. These biases could skew the perceived adoption rates, technical proficiency, and barriers. Future studies should incorporate observational methods or objective performance metrics (e.g., clinical audits) to validate self-reported findings. These limitations do not diminish the study's value but signal opportunities for additional investigation.

## 6. Conclusions and Recommendations

This study underscores the growing adoption of IOS in orthodontic practice across the Kurdistan region of Iraq, while outlining critical barriers that limit their full potential. Key findings include:

• Skill-device disparity: Many specialists lack technical proficiency in using IOS despite clinical adoption, largely due to insufficient formal training and reliance on self-directed learning.
• Adoption inequities: Private-sector practitioners and younger clinicians demonstrate higher IOS utilization compared to public-sector professionals and senior experts, reflecting resource disparities and generational resistance.
• Underutilized diagnostic potential: Current applications prioritize esthetic workflows (e.g., clear aligners) over advanced diagnostic capabilities, such as treatment planning and occlusal analysis.
• Structural barriers: High costs, limited training programs, and fragmented access to technology hinder widespread integration.

The first step toward a digital orthodontic revolution is not buying the device, but developing the skilled mindset to control it.

Recommendations:

To bridge these gaps and foster equitable, effective IOS integration, the following actionable strategies are proposed:

1. Curriculum reform:
  ⚬ Integrate mandatory IOS training into orthodontic academic programs, emphasizing technical proficiency (accuracy and software navigation) and diagnostic applications (arch analysis and surgical planning).
  ⚬ Establish competency-based certification programs to standardize expertise.
2. Financial accessibility:
  ⚬ Allocate government or international grants to subsidize IOS acquisition for public-sector clinics.
  ⚬ Partner with manufacturers (e.g., 3Shape and Medit) to offer leasing models or discounted pricing for rural practices.
3. Targeted skill development:
  ⚬ Conduct regional workshops led by global experts, tailored to:
    ▪ Senior practitioners: Focus on software updates and advanced workflows.
    ▪ Recent graduates: Align skills with modern clinical demands.
  ⚬ Develop free, multilingual e-learning platforms with Kurdish-language support.
4. Cross-sector collaboration:
  ⚬ Create public-private task forces to share resources, such as mobile training units for underserved areas.
  ⚬ Encourage joint research initiatives between universities and tech firms to design region-specific solutions (e.g., AI-driven diagnostic tools).
5. Awareness campaigns:
  ⚬ Launch a webinar series titled *‘Beyond Aligners: Unlocking IOS's Diagnostic Power'* to showcase underutilized applications.
  ⚬ Publish case studies demonstrating how IOS reduces treatment errors and long-term costs.
6. Policy advocacy:
  ⚬ Include ‘digital proficiency' as a licensing requirement for orthodontic practice.
  ⚬ Offer tax incentives to private clinics that provide free IOS training to public-sector professionals.

By implementing these recommendations, challenges can be transformed into opportunities to build a modern orthodontic system rooted in digital evidence, enhancing healthcare quality and positioning the region on the global medical innovation map. These are not merely reformative steps but an investment in the future of oral health for society.

## Figures and Tables

**Table 1:** Utilization of intraoral scanners.

Question	Responses	Frequency (%)
Usage of intraoral scanners in orthodontic work	Yes	61.03
	No	38.97

Duration of intraoral scanner usage	Less than 6 months	16.21
	6 months to 1 year	11.38
	1 to 2 years	31.04
	More than 2 years	41.37

Number of IOS devices in the office/clinic	1	81.03
	2	17.24
	3 or more	1.73

Frequency of IOS intraoral scanner usage in orthodontic practice	Daily	1.04
	Once a week	35.86
	Several times a week	5.53
	Once a month	38.96
	Several times a month	12.75
	Rarely	5.86
	Never	—

Participants' experience level with IOS and digital dentistry	Beginner	8.96
	Intermediate	75.17
	Advanced	13.11
	Expert	2.76

Perception of digital technology accuracy versus traditional casting	Strongly agree	5.86
	Agree	79.31
	Neutral	13.10
	Disagree	1.73
	Strongly disagree	—

Perception of future digital technology accuracy	Yes	91.37
	No	3.45
	Not sure	5.18

Software upgrade availability at no cost	Yes	92.41
	No	2.06
	Not sure	5.53

**Table 2:** Advantages and challenges.

Question	Responses	Frequency (%)
Enhancing patient comfort during orthodontic treatment with IOS scanners	Improved patient experience	48.96
	Reduced chair time	16.55
	Enhanced communication	32.07
	Other	2.42

Selecting key features of an intraoral scanner	Wireless capability	18.96
	Scan speed	24.82
	High-resolution imaging	28.62
	Easy software integration	5.86
	Lightweight and portable design	7.94
	Multicolor scanning	2.75
	Compatibility with third-party CAD/CAM systems	3.11
	User-friendly interface	4.14
	Autoclavable scanner tips for infection control	2.07
	Real-time feedback and visualization	1.73

Main challenges observed in utilizing intraoral scanners	Limited scanning accuracy	21.72
	High initial cost	35.17
	Difficulty in handling and maneuvering	5.52
	Limited compatibility with other software or systems	5.18
	Frequent need for maintenance and calibration	2.76
	Limited scanning depth or field of view	1.73
	Potential patient discomfort during scanning	3.79
	Concerns about data security and privacy	1.38
	Not applicable (I do not perceive any significant drawbacks)	22.75

**Table 3:** Correlation analysis of factors influencing IOS adoption and usage among orthodontists.

Topic	Variables	Correlation type	Correlation coefficient (95% CI; *n* = 290)	*p*-Value	Interpretation
Experience and usage	Age versus IOS usage	Pearson	**−0.42** (−0.52, −0.30)	0.002	Moderate negative correlation: Younger practitioners use IOS more frequently
	Age versus technological challenges	Spearman	**0.38** (0.25, 0.49)	0.021	**Moderate positive correlation**: Older individuals face more technological challenges

Training and knowledge	Public sector work versus lack of knowledge	Spearman	**0.65** (0.58, 0.71)	<0.001	**Strong positive correlation**: Public sector workers show a greater lack of knowledge
	Academic training versus clinical reliance	Spearman	**0.55** (0.47, 0.62)	<0.001	**Strong positive correlation**: Academic training increases reliance on scanners

Resources and challenges	Number of devices versus work challenges	Pearson	**−0.45** (−0.55, −0.33)	0.003	**Moderate negative correlation**: More devices are linked to fewer challenges

*Note: n*: Number of participating orthodontists; Pearson: Used when both variables are continuous and normally distributed; Spearman: Used when at least one variable is ordinal. Bold is used in correlation highlights the most critical correlation coefficients to draw reader attention, and in interpretation marks key concepts, followed by explanatory details.

Abbreviation: CI, Confidence Interval.

**Table 4:** Linear regression analysis of factors influencing the use of IOS in orthodontics.

Independent variable (predictors)	Dependent variable (outcome)	β (95% CI)	*p*-Value
Number of IOS devices in clinic	Monthly usage frequency	0.45 (0.20–0.70)	0.003
Clinical expertise (novice→expert)	Perceived accuracy of IOS technology	0.55 (0.40–0.70)	<0.001
Formal IOS training	IOS adoption in clinical practice	0.67 (0.50–0.84)	0.01
Free software upgrades	IOS adoption in clinical practice	0.32 (0.10–0.54)	0.04
Private sector employment (vs. public)	IOS adoption in clinical practice	0.32 (0.18–0.56)	0.03

*Note: β*, Standardized regression coefficient. Effect size interpretation: *β* < 0.10 (negligible), 0.10–0.29 (weak), 0.30–0.49 (moderate), ≥0.50 (strong).

Abbreviations: CI, confidence interval; IOS, intraoral scanner.

**Table 5:** Gap analysis between academic knowledge and practical application.

Domain	Academic knowledge (%)	Practical application (%)	Identified gaps
Experience	8.96% (formal IOS training)	61.03% use IOS clinically1.04% daily use 13.11% advanced expertise	Low practical expertise despite moderate adoption. Minimal daily integration, indicating superficial engagement.

Education	31.04% (academic IOS education)	57.24% see limited diagnostic impact 75.17% self-rate as “Intermediate expertise”.	Insufficient academic preparation on IOS technology Severe disconnect between academic training and practical technical proficiency.

Scanner characteristics	58.89% incorrect answers on technical terms	79.31% believe digital scans are more accurate than traditional methods	Overreliance on digital tools without foundational academic understanding. Low understanding of technical terms

Applications	11.72% affirm its diagnostic impact	5.52% diagnostic use 46.20% invisalign use	Limited diagnostic utilization despite perceived clinical benefits. Focus on cosmetic applications (e.g., Invisalign).

Challenges	60.54% (lack of knowledge/training in courses)	35.17% cite high cost as a barrier	High cost hinders widespread adoption and effective application. Poor foundational training during courses reduces technical proficiency and trust in IOS tools.

*Note:* Key questions driving the analysis include: Q 5, 8, 9, 17 (experience), Q 9, 14, 15 (education), Q 21, 27 (technical terms), Q 15, 18s (applications), and Q 20, 31(challenges).

## Data Availability

The corresponding author, Mohamad Radwan Sirri, can be contacted for any additional data or inquiries. E-mail: sirri.radwan@gmail.com. Mob: +963968702894.

## Nomenclature

IOS: Intraoral Scanners
CAD/CAM: Computer-Aided Design and Computer-Aided Manufacturing
SPSS: Statistical Package for the Social Sciences
CVR: Content Validity Ratio
CVI: Content Validity Index.

## Supporting Information

Supporting Information Table S1. Questionnaire development, validation, and reliability assurance.
