# Supplementary material for: Intraoral Scanners in Orthodontics: Utilization, Awareness, and Educational Implications Among Specialists in the Kurdistan Region, Iraq – A Cross-Sectional Study
Source: Int J Dent. 2025 Jun 4;2025:6663009. doi: 10.1155/ijod/6663009 (PMC12158578; doi:10.1155/ijod/6663009)
Supplement: Supporting Information — Table S1. Questionnaire development, validation, and reliability assurance. [file 6663009.f1.docx]

| **Supplementary Table 1:** Questionnaire Development, Validation, and Reliability Assurance | | | | |
| --- | --- | --- | --- | --- |
| **Aspect** | | **Details** | **Participants/Criteria** | **Results/Actions** |
| **Questionnaire Development** | |  |  |  |
| **1. Theoretical Foundation Phase** | Literature Review | Identification of core concepts related to IOS adoption in orthodontics | Authors | The initial draft of questions |
| **2. Validation Process Phase** | Expert Consultation | A comprehensive review of relevance, clarity, and completeness, with refinement for clinical accuracy and cultural appropriateness. | 6 experts (2 each from the Universities of Erbil, Dohuk, and Sulaymaniyah).  (+10 years of experience post-master’s degree) | Revised version of the questionnaire  (High validity of the questionnaire) |
|  | Statistical Measures | Calculation of CVR using Lawshe’s formula***** and CVR | Minimum acceptance: CVI ≥ 0.78 | Scientifically robust and highly valid questionnaire |
| **3. Reliability Assurance Phase** | Pilot Testing | Assessment of clarity, length, elimination of ambiguities, and alignment with real-world practice | 20 orthodontic specialists from diverse dental hospitals | Final version after modifications |
|  | Response Requirements | Electronically via Google Forms | Mandatory responses with specified single or multiple-choice options | Cronbach’s alpha = 0.85 (high reliability) |
| **CVR**: Content Validity Ratio; **CVI**: Content Validity Index.  ***:** Lawshe CH: A Quantitative Approach to Content Validity. Personnel Psychology. 2006, 28:563-575. 10.1111/j.1744-6570.1975.tb01393.x. | | | | |
